# Evaluation of the safety and efficacy of Zhenwu decoction as adjuvant therapy for the treatment of heart failure with reduced ejection fraction: A protocol for systematic review and meta-analysis

**DOI:** 10.1097/MD.0000000000028672

**Published:** 2022-01-28

**Authors:** Yue Han, Lanlin Huang, Guofu Zhong, Xiao Chang, Qinghua Zhu, Mujuan Xu, Chen Mingtai, Ling Men, Ling Wang

**Affiliations:** a Shenzhen Traditional Chinese Medicine Hospital, Shenzhen, Guangdong, China; b Guangzhou University of Chinese Medicine, Guangzhou, Guangdong, China.

**Keywords:** heart failure with reduced ejection fraction, protocol, systematic review, Zhenwu decoction

## Abstract

**Introduction:**

Heart failure with reduced ejection fraction (HFrEF) demonstrates a substanital threat to global public health. Several Chinese studies have been conducted to date evaluating the clinical efficacy of Zhenwu decoction (ZWD) as a treatment for HFrEF. The present systematic review will be conducted to more comprehensively evaluate the impact of ZWD on HFrEF outcomes.

**Methods:**

For this systematic review, all randomized controlled trials (RCTs) reporting on the effectiveness of ZWD as a treatment for HFrEF published as of December 30, 2021 in the Embase, PubMed, Springer, Web of Science, Cochrane Library, China Biomedical Literature Database, China National Knowledge Infrastructure, and the Wan-Fang databases will be identified without any language or publication restrictions. Two researchers will independently choose investigations, extract information, and gauge research quality. Primary outcomes of interest will include all-cause mortality and HF-associated mortality. Secondary outcomes will include the incidence of adverse events, ultrasonic cardiographic indices (including left ventricular ejection fraction and left ventricular mass index), New York Heart Association grade, N-terminal pro-b-type natriuretic peptide, B-type natriuretic peptide, and 6-minute walking distance. RevMan v 5.3 will be used to conduct meta-analyses where possible, with descriptive or subgroup analyses otherwise being conducted. Data will be given as risk ratios for categorical variables and mean difference for continuous variables.

**Results:**

This comprehensive protocol will aid in the systematic and objective evaluation of the efficacy and safety of ZWD as a treatment for HFrEF, providing a scientific basis for the clinical utilization of ZWD.

## 1 Introduction

Heart failure with reduced ejection fraction (HFrEF) is a syndrome wherein patients exhibit a reduced ejection fraction, heart failure (HF) symptoms, and aberrant systolic function in the presence or absence of normal diastolic function.^[1]^ Current guidelines define HFrEF as a diagnosis of HF with an EF ≤40%. HF was estimated to affect 5.7 million individuals from 2009 to 2012, rising to 6.5 million individuals from 2011 to 2014, of patients hospitalized for HF for the first time, 53% exhibited HFrEF of whom 70% were black males.^[2]^ Rates of HF vary significantly across Asian countries, affecting an estimated 4.5 million persons in China in 2014.^[3]^ Roughly one in 4 patients hospitalized for HF undergo readmission within 30 days, and this readmission rate is nearly 50% within a 6-month period.^[4]^ HF imposes a substantial burden on global healthcare systems, with a particularly disproportional impact on low- and middle-income nations in South America, Eastern Europe, Africa, and the Asia-Pacific region where rates of HF are rising rapidly along with other cardiovascular conditions including diabetes mellitus and hypertension.^[5]^ Treatments for HF include the implantation of devices capable of regulating cardiac activity and pharmacological intervention.^[6]^ Even with these interventions, however, HF is incurable and patients generally exhibit high mortality rates and a poor prognosis.^[1]^ There is thus an urgent need to develop novel therapies for affected patients.

Complementary and alternative medicine (CAM) is an area of growing research interest for HFrEF treatment, with several systemic reviews and meta-analyses having been conducted for the exploration of the efficacy of different CAM agents in the treatment of this condition by evaluating results from extant randomized controlled trials (RCTs).^[7]^ Chinese herbal medicines are a form of CAM therapy that have been employed for the treatment of HFrEF-related conditions in China for at least 2500 years. Zhenwu decoction (ZWD) is a traditional herbal preparation employed in traditional Chinese medicine that was formulated by the physician Zhongjing Zhang in *Shang Han Lun* (Treatise on Febrile and Miscellaneous Diseases) roughly 18 centuries ago. It is composed of Poria (Fu Ling, *Scierotium Poriae Cocos*), processed aconite (Fu Zi, *Radix Lateralis Praeparata Aconiti Carmichaeli*), White Peony Root (Bai Shao, *Radix Albus Paeoniae Lactiflorae*), White Atractylodes Rhizome (Bai Zhu, *Rhizoma Atractylodis Macrocephalae*), and fresh ginger (Sheng Jiang, *Rhizoma Zingiberis Recens*). In prior pharmacological analyses, ZWD was shown to promote p-ERK5 upregulation within cardiomyocytes while suppressing mitochondrial autophagic gene expression,^[8]^ thereby aiding in HF treatment. Several other studies to date have also explored the clinical potential of ZWD as a treatment for HFrEF among Chinese patients.^[9,10]^ To date, however, there have been no meta-analyses that summarize these results, with several questions remaining regarding the benefits of such treatment. This study will thus be conducted with the following goals:

1. to assess the relative efficacy of ZWD as compared to nontreatment, placebo treatment, or the treatment with anti-HF drugs;
2. to gauge the effectiveness of ZWD along with anti-HF medications relative to anti-HF medications alone; and
3. to establish the safety of ZWD.

## 2 Methods

This meta-analysis was registered at Open Science Framework registries (OSF, https://osf.io/9fty3) with a registration number of DOI: 10.17605/OSF.IO/9FTY3. and will be executed as per the Preferred Reporting Items for Systematic Reviews and Meta-Analyses Protocols statement instructions.^[11]^

### 2.1 Study

RCTs eligible for inclusion will be English or Chinese RCTs evaluating the use of ZWD for the treatment of HFrEF. Medical record reports and semi-random RCTs will be excluded from this study.

### 2.2 Participants

Participants eligible for inclusion in this study will include individuals diagnosed with HFrEF (New York Heart Association class IV or II) over 18 years of age without any gender, course, or comorbidity limitations. Patients with acute or chronic HFrEF will be eligible for inclusion, with patients that have undergone cardiac transplantation, cardiac arrest, or cardiac shock being excluded.

### 2.3 Interventions

Control cohorts will include Western medicine protocols, while interventional cohorts will include ZWD treatment protocols. There will be no limitations on ZWD dosage, treatment, or manufacturer.

### 2.4 Outcome measures

#### 2.4.1 Primary outcomes

∘ All-cause mortality
∘ HF-related mortality

#### 2.4.2 Secondary outcomes

∘ Ultrasonic cardiograph indices (e.g., left ventricular mass index, left ventricular ejection fraction)
∘ New York Heart Association grade
∘ B-type natriuretic peptide
∘ N-terminal pro-b-type natriuretic peptide
∘ Six-minutes walking distance
∘ Adverse events

### 2.5 Search strategy

The present systematic review will consist of the manual and electronic searching for RCTs pertaining to the use of ZWD for the treatment of HFrEF published as of December 30, 2021. No publication or language limitations will be imposed on this analysis. Analyzed databases will comprise Embase, PubMed, Springer, Web of Science, the Cochrane Library, the China Biomedical Literature Database, the China National Knowledge Infrastructure, and the Wan-Fang Database using the search phrases: Zhenwu decoction and heart failure. This same strategy will also be used to assess Chinese databases. The final strategy of the search will be formulated as per the guidelines of the Cochrane Handbook. The search strategy of PubMed is listed in Table 1.

**Table 1:** Literature-search strategy employed for PubMed repository.

Number	Search terms
#1	Heart failure [MeSH]
#2	Heart failure [Title/Abstract]
#3	#1 OR #2
#4	Zhenwu Decoction [MeSH]
#5	Zhenwu Decoction [Title/Abstract]
#6	ZWD [Title/Abstract]
#7	#4 OR #5 OR #6
#8	Randomized controlled trial [Title/Abstract]
#9	Clinical study [Title/Abstract]
#10	Controlled study [Title/Abstract]
#11	#8 OR #9 OR #10
#12	#3 AND #7 AND #11

In progress trials with unpublished information will be retrieved from registries including the National Institute of Health, the International Clinical Trials Registry Platform (http://www.who.int/ictrp/en/), the Chinese clinical registry (http://www.chictr.org/en/), ClinicalTrials.gov (https://www.clinicaltrials.gov/), and the Australian New Zealand Clinical Trials Registry (http://www.anzctr.org.au/). References for published reviews will also be searched using appropriate retrieval methods. Efforts will be made to contact the corresponding authors of investigations for which data are incomplete.

### 2.6 Study selection

**Figure 1:**
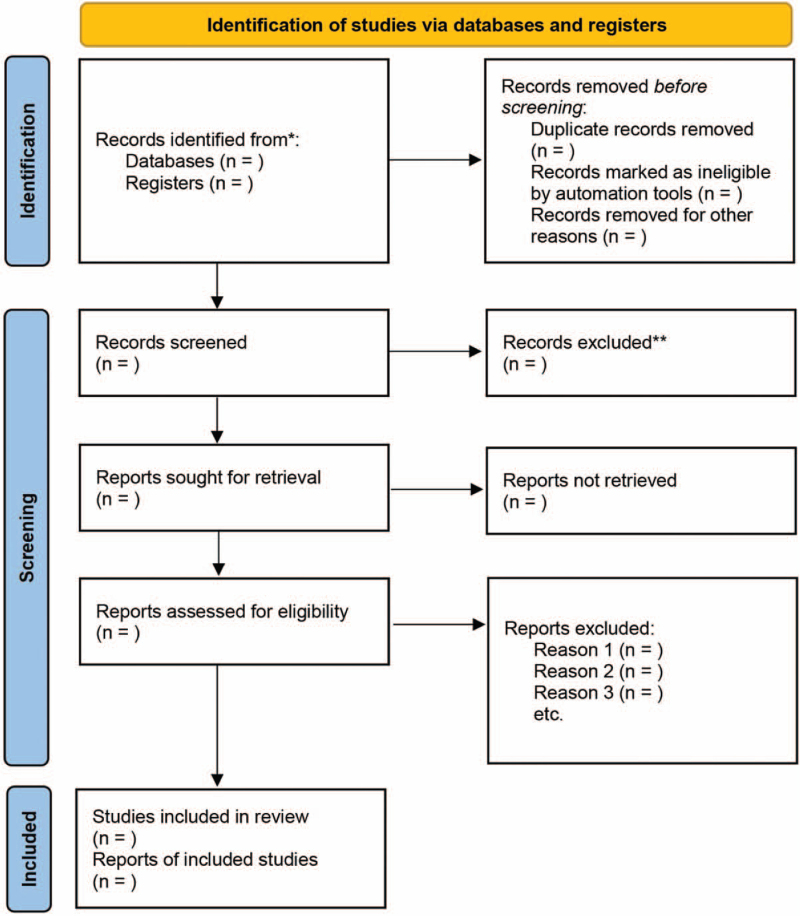
Flow diagram of study selection process.

Titles and abstracts for identified studies retrieved via the above search will be independently evaluated by 2 investigators who will exclude those investigations not meeting with the criteria of eligibility. The full text will be retrieved for the remaining trials, which will be evaluated for eligibility with the aid of 2 independent reviewers. Trial exclusion rationale at the full-text level will be documented, and through discussion or consensus with a fifth investigator, discrepancies will be resolved. The study selection procedure is outlined in Figure 1.

### 2.7 Data extraction

A standardized form will be independently used by 2 investigators to extract data including article title, first or corresponding author, country, journal, year of publication, institution, trial setting, sponsor, inclusion/exclusion criteria, trial design specifics (blinding, randomization, allocation concealment), patient characteristics (age, gender, comorbidities, HFrEF severity and course), sample size, intervention and control treatment details (including dosage, ZWD type and composition, duration of treatment, and co-interventions), outcome details (time points, mean values, response/non-response events, and mean difference values), and other factors with the potential to aid in bias detection (termination time, data for analysis, register ID). The division of labor will be identical to that in the section of Study Selection, with a fifth investigator aiding in the resolution of disagreements.

### 2.8 Risk of bias analyses

The bias risk and methodological quality for included investigations will be evaluated in compliance with the Cochrane Handbook for Systematic Reviews of Interventions, v 6 risk of bias 2.0 (ROB2.0) gadget.^[12]^ These analyses will be independently performed by one investigator and confirmed by another, with a third investigator serving to resolve any discrepancies.

### 2.9 Data synthesis and analyses

RevMan V 5.3 will be employed to synthesize and scrutinize included information. Continuous data will be assessed based upon mean difference values with 95% CIs, while categorical data will be analyzed using risk ratios with 95% CIs. Potential heterogeneity will be assessed as per the Cochrane Handbook for Systematic Reviews of Interventions based on a visual inspection of forest plots, heterogeneity χ^2^ tests, and Higgins’ *I*^2^ statistic. Data will be pooled and analyzed using fixed-effects models if *I*^*2*^ is < 50% and *P* > .1, whereas random-effects models will otherwise be employed. Meta-analyses with random-effects models will be implemented to gauge overall treatment efficacy when heterogeneity is observed, but meta-analyses will not be performed when there is considerable heterogeneity among trials, with a qualitative summary instead being provided. Subgroups will be considered for subgroup analyses as appropriate.

### 2.10 Additional analyses

#### 2.10.1 Subgroup analyses

Subgroup analyses will be performed for available data when factors have the potential to influence interventional outcomes. Potential subgroups of interest will include gender, age, control type (active treatments, sham treatments, or untreated/waiting list participants), follow-up duration (short term [2 months or less] vs long term [>2 months]). Causes of identified heterogeneity will be further interpreted in the discussion section.

#### 2.10.2 Sensitivity analyses

Sensitivity analyses will be employed to gauge the reliability and robustness of our results based upon potential methodological weaknesses (such as a lack of adequate allocation concealment or sequence generation), missing data, or sample sizes (more than or less than 30 individuals per group). Risk of bias will be further defined when sensitivity analyses reveal low robustness. Those studies with an unclear or high risk of bias will be excluded from all analyses.

### 2.11 Publication bias analyses

Funnel plots and Begg test will be conducted using Stata 14 to gauge potential publication bias, with a *P* > .05 being indicative of a lack of publication bias.

### 2.12 Quality of evidence analyses

The evidence quality for the obtained results will be assessed based on the Grades of Recommendation, Assessment, Development, and Evaluation^[13]^ method. Analyzed factors will include risk of bias, publication bias, imprecision, and indirectness, with potential assessments including very low, low, moderate, and high.

## 3 Discussion

Several reports^[9,10]^ have indicated that ZWD treatment can affectively alleviate HFrEF symptoms without inducing significant side effects. No systematic reviews regarding the efficacy of this therapeutic approach, however, have yet been published. There is thus a clear need for such a comprehensive overview, and the analysis proposed herein will consist of 4 key sections: study selection, inclusion, data extraction, and synthesis. Limitations of this protocol include the potential for heterogeneity among studies owing to variations in evaluative criteria and ZWD dosing. The overall goal of this analysis is to provide an evidence-based foundation for clinicians seeking to make more accurate decisions pertaining to HFrEF patient treatment.

## Acknowledgments

The authors would like to thank all the reviewers who participated in the review and MJEditor (www.mjeditor.com) for its linguistic assistance during the preparation of this manuscript.

## Author contributions

**Conceptualization:** Chen Mingtai.

**Data curation:** ling Wang, Lanlin Huang, Chen Mingtai.

**Formal analysis:** Lanlin Huang.

**Investigation:** Ling Men.

**Methodology:** Guofu Zhong, Ling Men.

**Project administration:** Guofu Zhong.

**Resources:** Xiao Chang.

**Software:** Xiao Chang.

**Supervision:** Qinghua Zhu, Mujuan Xu.

**Validation:** Qinghua Zhu.

**Visualization:** Mujuan Xu.

**Writing – original draft:** Yue Han.

**Writing – review & editing:** ling Wang.
